# Quiet Quitting of Job in Nurses Working in Hospitals, a Multicenter Study in Iran

**DOI:** 10.1002/brb3.71439

**Published:** 2026-04-29

**Authors:** Sulmaz Ghahramani, Ahmad Nemati, Mahdi Hazrati, Mobin Gholami, Aida Eskandari, Hassan Gholami, Kamran Bagheri Lankarani

**Affiliations:** ^1^ Health Policy Research Center Shiraz University of Medical Sciences Shiraz Iran; ^2^ Faculty of Medicine Mashhad University of Medical Sciences Mashhad Iran; ^3^ Department of Health Vice‐Chancellor of Hamedan University of Medical Sciences Hamedan Iran; ^4^ Healthcare Services Management, School of Health Hamadan University of Medical Sciences Hamadan Iran; ^5^ Department of Medical Education, Faculty of Medicine Mashhad University of Medical Sciences Mashhad Iran

**Keywords:** hospital, Iran, nurses, quiet quitting, scale, work place

## Abstract

**Introduction:**

Quiet quitting (QQ), characterized by reduced work engagement and effort, is an emerging concern in healthcare, potentially impacting patient care and organizational outcomes. This study aims to investigate the prevalence and associated factors of QQ among Iranian nurses.

**Methods:**

A 2024 cross‐sectional survey was conducted in thirteen hospitals located in the Iranian cities of two provinces in Iran. Nurses were randomly selected by using stratified sampling from various hospital departments. The nine‐item QQ scale was adapted and validated for use within the Iranian healthcare environment. Receiver Operating Characteristic (ROC) curve analysis led to the identification of an optimal quiet quitting scale (QQS) cutoff point of 2.38. To determine key predictors of QQ, logistic regression analysis was employed.

**Results:**

The study included 754 participants (541 females and 213 males) with an average age of 33.17 ± 7.32 years. The prevalence of QQ was 59.5% among Iranian nurses. Among QQS subscales, lack of motivation had the highest average score, while detachment had the lowest mean score. Logistic regression analysis indicated that nurses employed in specialized hospitals and those experiencing higher levels of job burnout had increased odds of engaging in QQ (2.249, 95% CI: 1.581, 3.199, *p* < 0.001 and 1.49, 95% CI: 1.3, 1.709, *p* < 0.001, respectively). In contrast, higher job satisfaction was associated with lower odds of QQ (0.747, 95% CI: 0.586, 0.954, *p* = 0.01).

**Conclusion:**

We found the QQ Scale as a valid and reliable tool for nurses in healthcare settings of Iran. Findings demonstrated QQ was prevalent among Iranian nurses and emphasize the role of organizational aspects, particularly hospital type, and individual well‐being elements, such as job satisfaction and burnout, in shaping QQ behavior among nurses. Further research is warranted to explore additional factors contributing to QQ and to develop effective strategies for enhancing nurse engagement.

## Graphical abstract

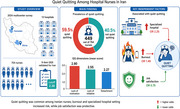

## 1 Introduction

“Quiet quitting (QQ) or silent resignation” refers to the intentional practice of performing only the minimum required work in a job while avoiding additional responsibilities or extra‐role behaviors (Boy and Sürmeli 2023; Nguyen 2024; Formica and Sfodera 2022). In this context, employees meet core job requirements but refrain from taking on tasks beyond their formal duties (Boy and Sürmeli 2023; Formica and Sfodera 2022).

The term gained widespread attention during and after the COVID‐19 pandemic, alongside heightened concerns about work–life balance (Nguyen 2024; Scheyett 2023). QQ appears to function as a defense mechanism for individuals who, despite needing their current income, feel dissatisfied with their job and compensation (Scheyett 2023; Karrani et al. 2024). Furthermore, the phenomenon is rooted in three core dimensions: a lack of initiative, lack of motivation, and detachment (Boy and Sürmeli 2023; Galanis et al. 2023).

While remote work was not feasible in healthcare during and after the pandemic, healthcare workers were similarly influenced by this trend (Boy and Sürmeli 2023; Kang et al. 2023; Pevec 2023). In healthcare, factors contributing to this phenomenon include organizational and workload‐related stressors (e.g., staffing shortages, long shifts, heavy workload, and stressful work environments), occupational risks (e.g., exposure to infection and workplace violence), and inadequate compensation (Boy and Sürmeli 2023; Kang et al. 2023; Azizi et al. 2024; Volosovets et al. 2022; Arneja and Gilardino 2023; Ghahramani et al. 2024). These factors have been found to be more severe among nurses, and it is expected that prevalence of QQ is higher among nurses than other healthcare staff (Galanis et al. 2024). Nurses constitute a large segment of the hospital workforce and play a central role in care delivery and healthcare system functioning (Davis et al. 1996). Despite the fact that QQ may play as a defense mechanism for nurses, it would lead to detrimental consequences on healthcare system performance, nurses, and health status of people (Nguyen 2024; Kumar 2023). QQ may contribute to reduced quality of care and lower organizational commitment, potentially increasing system costs and decreasing effectiveness, while also negatively affecting patient satisfaction and outcomes (Nguyen 2024; Kumar 2023; Johar et al. 2023).

As mentioned earlier, QQ might be more prevalent among nurses in comparison to other healthcare professions, and this hypothesis was confirmed by former studies, as > 67% of nurses in Greece reported QQ more than other healthcare staff (Galanis et al. 2024). In Greece's study, job‐related factors, burnout, job satisfaction, job turnover, violence, demographic characteristics, and other factors had an impact on QQ status among nurses (Galanis et al. 2024). In line with many countries, Iranian healthcare workers face substantial challenges, including burnout, job dissatisfaction, and low motivation (Ghahramani et al. 2024; Ghahramani et al. 2022; Ghahramani et al. 2023). National surveys suggest that more than half of Iranian nurses report job dissatisfaction and burnout, which worsened during the COVID‐19 pandemic (Hajebi et al. 2022; Kamali et al. 2020). High turnover intentions, including an increasing desire to immigrate, have been reported among Iranian nurses, along with workplace violence (Ghahramani et al. 2022; Amiri et al. 2025; Dalvand et al. 2018; Mohtashami et al. 2025). Mental health concerns are also common, with prior research indicating elevated levels of depression and anxiety among Iranian nurses (Hemmati et al. 2021; Nemati et al. 2025). These struggles are compounded by low wages and inadequate benefits, underscoring the financial strain experienced by nurses (Payscale 2025; Shamsi and Peyravi 2020). Collectively, these conditions have contributed to public protests and strikes among nurses, reflecting demands for improved working conditions and livelihoods. Yet, many of these demands remained unmet, sustaining a climate of dissatisfaction at work (Saki et al. 2024). Accordingly, QQ may constitute an important and potentially prevalent issue among Iranian nurses, although it has not yet been empirically documented in the peer‐reviewed literature.

Given the relative novelty of the QQ, empirical studies across diverse settings are needed to clarify its scope and correlates among healthcare workers. Obviously, the factors contributing to QQ can vary in each country, since cultural context, demographic background, health care system, economic status, and work environment are different. Accordingly, measurement properties of QQ instruments (e.g., reliability, validity, and cut‐off thresholds) may also vary across languages and cultural contexts. This study aims to investigate the prevalence of QQ among nurses in university hospitals in Fars and Hamadan provinces, exploring its relationship with job burnout, job satisfaction, and selected occupational and demographic variables. Moreover, we evaluated the psychometric properties (validity and reliability) of the Iranian version of QQ questionnaire scale.

## 2 Methods

This cross‐sectional study investigated QQ among nurses working in public and university hospitals in two cities, Hamadan and Shiraz, Iran, during 2024. Both cities are the centers of two large provinces in the south and west of Iran.

### 2.1 Participants

Nurses were recruited from a total of 13 public and university‐affiliated hospitals across Hamadan and Shiraz.

Inclusion criteria were (a) currently employed as a registered nurse at one of the participating hospitals and (b) a minimum of 6 months of employment in their current position within the hospital.

Exclusion criteria were (a) nurses employed on a part‐time basis and (b) nurses who indicated dissatisfaction with participating in the study.

A sample size of 400 nurses per city (total *n* = 800) was determined using the Cochrane formula, assuming a 60% prevalence of QQ among nurses based on the most comparable prior study, with a 95% confidence level and a 5% margin of error (Galanis et al. 2024). Within each city, hospitals were selected to ensure representation of seven general and six specialized hospitals: Shiraz (seven hospitals) and Hamadan (six hospitals). Sampling within each hospital was proportional to the number of beds and was stratified by gender, employment status (permanent or contract), and work area (emergency department, intensive care units, and inpatient wards) to ensure a representative sample. Nurses working rotating and fixed shifts were included. Sampling was monitored to ensure adherence to the sampling protocol.

### 2.2 Data Collection

Data were collected through face‐to‐face, interviewer‐administered questionnaires covering demographic variables, occupational characteristics, and the nine‐item QQ. Each interview lasted approximately 10–15 min. This approach was selected to reduce nonresponse and response bias, as nurses experiencing QQ may be less likely to complete self‐administered (e.g., online) questionnaires outside working hours. In addition, conducting interviews onsite during working hours facilitated participation and improved completion rates. The interviews were conducted using a standardized questionnaire comprising the following sections:

#### 2.2.1 Questionnaires and Other Measurement Tools

*Demographic information* : In this section nurses were asked about age, gender, marital status, and educational level (diploma and upper diploma, bachelor's, master's, doctorate).

*Occupational characteristics*: The city of the study and the type of hospital (general vs. specialized hospital) were evaluated. In this section, work history (years) and current service area within the hospital (emergency department, intensive care unit, and inpatient wards) were assessed. Moreover, trained interviewers asked about monthly working hours, work shift patterns (fixed or rotating), and history of workplace violence within the past 3 months. Nurses were also asked about recent desire about emigration within the past 3 months and intention to change occupation within the past 3 months (job turnover). Furthermore, engagement with family and friends was assessed by asking, “Do you spend adequate time with your family members or friends?”

*Job satisfaction*, defined as a positive emotional state resulting from the evaluation of one's work experiences (Heslop et al. 2002). A single‐item measure was used to assess overall job satisfaction. They were asked, “Are you satisfied with your job?” a 5‐point Likert scale ranging from 1 (*strongly disagree*), 2 (*relatively disagree*), 3 (*not satisfied or dissatisfied*), 4 (*relatively agree*), and 5 (*strongly agree*) (Heslop et al. 2002; Shanafelt et al. 2009). Participants were classified as dissatisfied if they selected a response of 1–3, indicating dissatisfaction with their job.

*Job burnout*, a condition of emotional, physical, and mental exhaustion caused by prolonged stress (Ghahramani et al. 2024; Hansen and Pit 2016). This variable was assessed using a single item burnout. They were asked to share their opinions about a question with a 7‐point time Likert scale, from “*never*” scoring 0 to “*everyday*” scoring 6 (Ghahramani et al. 2024; Hansen and Pit 2016). The sentences were “I feel burned out with my job.” It was considered positive if they answered the question more than “once a week” with a score of 4 or more frequently than once a week.

#### 2.2.2 QQ Scale and Its Validity and Reliability Assessment

In this study, we used the nine‐item quiet quitting scale (QQS) developed by Galanis and colleagues (Galanis et al. 2023), which has demonstrated acceptable validity and reliability in previous studies conducted outside Iran. The QQS comprises three subscales: detachment (four items), lack of initiative (three items), and lack of motivation (two items). Participants rated their agreement with each item on a 5‐point Likert scale ranging from 1 (*strongly disagree*) to 5 (*strongly agree*) (Galanis et al. 2023); Questions 5 and 7 were about frequency (“How often”?) Likert scale ranging from 1 (*never*) to 5 (*always*). The mean score across all nine items was calculated to provide an overall QQ score, ranging from 1 to 5, with higher scores indicating higher levels of disengagement. In addition, Questions 7, 8, and 9 are scoring reversely. The prior study conducted in Greece used a cut‐off of 2.06 to define job disengagement or QQ (Galanis et al. 2023). Given the potential for cultural and language differences in interpreting the QQS and variations in the work environment of Iranian nurses, we evaluated the appropriateness of the QQ scale among Iranian nurses. (Boy and Sürmeli 2023) Psychometric properties, including face validity, content validity, concurrent validity, and reliability, were evaluated for the QQS.

A panel of eight experts assessed the face validity of the QQS by evaluating the relevance, clarity, and difficulty of each item. Content validity was assessed by a group of 10 experts with backgrounds in epidemiology, biostatistics, nursing practice, hospital management, head nursing, community medicine, and public health. The Content Validity Index (CVI) was used to evaluate the relevance of the questions based on a four‐point scale (1 = *not relevant*, 2 = *needs essential revision*, 3 = *relevant but needs revision*, and 4 = *very relevant*). The CVI was calculated by dividing the number of respondents who scored three or four by the total number of respondents. A score below 0.70 indicates that the item is unacceptable and should be removed.

We assessed concurrent validity using the Job Satisfaction Survey (JSS), the single item burnout measure, and a single‐item measure for job turnover intention. We calculated 1 − specificity, sensitivity, and Youden's J index ([sensitivity + specificity] − 1), which indicates the overall effectiveness of both sensitivity and specificity. Values closer to 1 indicate greater effectiveness (Youden 1950).

Receiver operating characteristic (ROC) curves, illustrating sensitivity versus (1 − specificity), were used to determine the optimal trade‐off between sensitivity and specificity compared with a reference standard (Wallace et al. 2006). Because the primary purpose of the QQS in this study was to identify nurses at risk of QQ for potential monitoring and early intervention, we prioritized higher sensitivity over specificity when selecting the cut‐off value. In practical terms, this meant favoring a threshold that minimized false negatives (nurses with relevant problems incorrectly classified as non‑QQ), while accepting a moderate number of false positives, in line with recommendations for screening tools (Lalkhen and McCluskey 2008; Ghahramani et al. 2025). We calculated the area under the ROC curve (AUROC) with a 95% confidence interval (CI) to compare the overall performance of the QQS. Statistical tests within the ROC curve framework evaluated whether the model possesses statistically significant discriminatory power and whether the observed differences between models are non‐random. In the context of burnout, job satisfaction, and intention to leave, these tests examined the extent to which the QQS score effectively differentiates nurses experiencing these conditions from those who do not, thereby assessing its validity as a predictive measure in occupational settings.

### 2.3 Data Analysis

Statistical analyses were performed by using SPSS version 27. Descriptive statistics (frequencies, percentages, means, and standard deviations) were used to summarize epidemiological participant characteristics, work shifts, work hours, QQ score, job satisfaction levels, intention of job turnover, violence, desire for immigration, and job burnout scores. Association between categorical variables (e.g., demographic characteristics, occupational characteristics) and QQ (yes/no) were analyzed using the chi‐square test. A significance level of *p* < 0.05 was used for all statistical tests.

Logistic regression analysis was performed using the ENTER method, in which all selected predictor variables were entered into the model simultaneously in order to examine factors associated with nurses with reporting QQ and without QQ. Epidemiological characteristics, occupational characteristics, job satisfaction, and job burnout were entered as independent variables in the regression models, with QQ score as the dependent variable.

### 2.4 Ethical Considerations

Prior to the interview, the study's purpose was explained to each participant, and written informed consent was obtained. Moreover, they were informed about their right to withdraw from this study if they want. The study protocol was approved by the ethics committee of Shiraz University of Medical Sciences, encoded IR.SUMS.REC.1402.583 and it was conducted in accordance with Helsinki the Declaration of Helsinki. Additionally, their identities remained confidential and were converted into codes.

## 3 Results

Our study included 754 nurses, 368 of them working in Shiraz and 386 of them in Hamedan. In order to evaluate QQ scale and define the best appropriate cut‐off for it in Iranian context, following steps were taken. Internal consistency reliability was measured using Cronbach's alpha, with an alpha value of 0.6 or higher considered acceptable.

The QQS demonstrated acceptable psychometric properties. In the face validity assessment, expert opinions were collected, and none of the items were removed. The CVI score in the content validity assessment was 0.89, indicating acceptable validity (Supporting Information).

The nine‐item QQ Scale showed an AUROC of 0.61 (0.56–0.65) for job turnover intention, 0.69 (0.64–0.74) for burnout, and 0.67 (0.64–0.71) for job satisfaction, all with *p* < 0.001, indicating a significantly high performance (Supporting Information). Cronbach's alpha for the QQS was 0.66.

Sensitivities, specificities, and Youden's J indices for different tools are displayed in Table 1. According to Youden's J index, 2.38 was the optimal cutoff for detecting QQ in the QQS. However, Youden's J index should be interpreted with caution, as sensitivity and specificity are equally weighted in its calculation. When using QQS, sensitivity and specificity are not always regarded as equally important. It appears that sensitivity is generally prioritized over specificity (Lalkhen and McCluskey 2008; Barnard‐Brak et al. 2016).

**TABLE 1:** Performance of nine items QQS for detecting burn out, job satisfaction and job turn over intention (*N* = 754).

Criterion	Cut‐off	Sensitivity	1 − specificity	Youden's *J* index
Job turnover intention	2.38	0.637	0.493	0.507
Burn out	2.38	0.654	0.323	0.677
Job satisfaction	2.38	0.674	0.427	0.573

The final QQS cutoff of 2.38 was chosen as the point that maximized Youden's J index while maintaining high sensitivity, consistent with our goal of minimizing under‑identification of at‑risk nurses.

As can be seen from Table 2, the sample consisted predominantly of female nurses (71.8%) with a mean age of 33.17 years (SD = 7.32), with the largest age group being 20–29 years old (40.2%). Moreover, majority of them were married (57.6%).

**TABLE 2:** Socio‐demographic characteristics of the nurses (*N* = 754).

Variable	Subgroup	N (percentage of each subgroup among all participants)	Prevalence of quiet quitting in each subgroup		*p*‐value
			No, *N* (%)	Yes, *N* (%)	
**Age (mean ± SD –33.17 ± 7. 32)**	20–29	303 (40.2)	100 (33)	203 (67)	**< 0.001**
	30–39	284 (37.7)	120 (42.3)	164 (57.7)	
	40–49	151 (20)	74 (49)	77 (51)	
	≥ 50	16 (2.1)	11(68.8)	5 (31.2)	
**Gender**	Female	541 (71.8)	220 (40.7)	321 (59.3	0.848
	Male	213 (28.2)	85 (39.9)	128 (60.1)	
**Education**	Diploma bachelor's or degree or associate	685 (90.8)	269 (39.3)	416 (60.7)	**0.037**
	Masters and above	69 (9.2)	36 (52.2)	33 (47.8)	
**Marital status**	Single/divorced/widowed	320 (42.4)	118 (36.9)	202 (63.1)	0.086
	Married	434 (57.6)	187 (43.1)	247 (56.9)	
**Total**			305	449	

p‐value is less than 0.05 (p < 0.05).

Most nurses worked rotating shifts (89.7%) and were employed in general hospitals (65.5%) (Table 3). The distribution across departments was as follows: Emergency (16.8%), ICU/CCU (30.8%), and Admission (52.4%). The mean clinical experience was 9.19 ± 6.99 years, with a mean monthly work hour of 204.49 ± 51.99. A substantial portion of nurses reported experiencing violence in the workplace (61.4%), and over half had considered immigration (57.7%) (Table 3). A large percentage of nurses expressed job turnover intention (71%). Furthermore, 82.4% of participants reported job burnout, while 31.7% of them were satisfied with their jobs.

**TABLE 3:** Work characteristics of the nurses (*N* = 754).

Variable	Subgroup	N (percentage of each subgroup among all participants)	Prevalence of quiet quitting in each subgroup		*p*‐value
			No, *N* (%)	Yes, *N* (%)	
**Type of shift**	Rotating shifts	676 (89.7)	269 (39.8)	407 (60.2)	0.278
	Fixed shifts	78 (10.3)	36 (46.2)	42 (53.8)	
**Hospital type**	General	494 (65.5)	222 (44.9)	272 (55.1)	**< 0.001**
	Specialized	260 (34.5)	83 (31.9)	177 (68.1)	
**Clinical experience (years) (mean ± SD – 9.19 ± 6.99)**	≤ 10	483 (64.1)	169 (35)	314 (65)	**< 0.001**
	> 10	271 (35.9)	136 (50.2)	135 (49.8)	
**Monthly work hours (mean ± SD – 204.49 ± 51.99)**	< 200	390 (51.7)	183 (46.9)	207 (53.1)	**< 0.001**
	≥ 200	364 (48.3)	122 (33.5)	242 (66.5)	
**Job tenure in current department (years) (mean ± SD – 4.80 ± 4.54)**	≤ 4	443 (58.8)	161 (36.3)	282 (63.7)	**0.006**
	>4	311 (41.2)	144 (46.3)	167 (53.7)	
**Department**	Emergency	127 (16.8)	44 (34.6)	83 (65.4)	0.313
	ICU/CCU	232 (30.8)	94 (40.5)	138 (59.5)	
	Admission	395 (52.4)	167 (42.3)	228 (57.7)	
**Experience of violence in workplace**	No	291 (38.6)	139 (47.8)	152 (52.2)	**0.001**
	Yes	463 (61.4)	166 (35.9)	297 (64.1)	
**Thought of emigration**	No	319 (42.3)	150 (47)	169 (53)	**0.002**
	Yes	435 (57.7)	155 (35.6)	280 (64.4)	
**Job turnover intention**	No	219 (29)	111 (50.7)	108 (49.3)	**< 0.001**
	Yes	535 (71)	194 (36.3)	341 (63.7)	
**Family and friend engagement**	No	667 (88.5)	253 (37.9)	414 (62.1)	**< 0.001**
	Yes	87 (11.5)	52 (59.8)	35 (40.2)	
**Burnout (mean ± SD – 4.71 ± 1.30)**	No (≤ 3)	133 (17.6)	90 (67.7)	43 (32.3)	**< 0.001**
	Yes (> 3)	621 (82.4)	215 (34.6)	406 (65.4)	
**Job satisfaction (mean ± SD – 2.62 ± 0.76)**	Not satisfied (1–3)	515 (68.3)	168 (32.6)	347 (67.4)	**< 0.001**
	Satisfied (4–5)	239 (31.7)	137 (57.3)	102 (42.7)	
**Total**			305	449	

p‐value is less than 0.05 (p < 0.05).

Based on a cutoff score of 2.38 on the QQ scale (Table 1), 59.5% (*n* = 449) of the nurses were classified as engaging in QQ behaviors (scoring 2.38 or higher), while 40.5% (*n* = 305) were not. The average score of QQ sale was 2.50 ± 0.55 (Table 4). In terms of the average domains, lack of motivation and detachment had the highest and the lowest mean scores, respectively. Additionally, the average of scores in each domain among quiet quitters and non‐quiet quitters are compared in Table 4.

**TABLE 4:** Mean and SD of each subscales in quiet quitters and non‐quiet quitters groups.

Quiet quitting scale	Quiet quitting score (mean ± SD)		
	Overall score	Score among non‐quiet quitters	Score among quiet quitters
Total	2.50 ± 0.55	1.99 ± 0.32	2.85 ± 0.36
Detachment	2.27 ± 0.66	1.80 ± 0.51	2.58 ± 0.56
Lack of initiative	2.55 ± 0.74	2.03 ± 0.55	2.91 ± 0.63
Lack of motivation	2.90 ± 1.0	2.28 ± 0.81	3.30 ± 0.90

Association of QQ with demographic variables is also presented in Table 2. Significant differences in QQ prevalence were observed between nurses working in Shiraz (51.6%) and Hamadan (67.1%) (*p* < 0.001). The younger age group (20–29 age group) was associated with higher prevalence of QQ in comparison with older age groups. Moreover, lower educational level was significantly associated with QQ (*p* = 0.037).

Table 3 also presents that nurses working in specialized hospitals had a higher prevalence of QQ (68.1%) compared to those in general hospitals (55.1%). Additionally, fewer clinical experience was significantly associated with QQ (65% vs. 49.8%), but more monthly work hour was significantly associated with QQ (53.1% vs. 66.5%). Nurses with job tenure of ≤ 4 years reported a higher prevalence of QQ (63.7%) compared to those with > 4 years of tenure (53.7%) (*p* = 0.006). Those who had adequate time to hang out with friends and family had the lowest prevalence of QQ (*p* < 0.001). Finally, nurses experiencing violence in the workplace (64.1% vs. 52.2%), having emigration thoughts (64.4% vs. 53%), having job turnover intention (63.7% vs. 43.9%), experiencing job burnout (65.4% vs. 32.3%), and dissatisfaction with their jobs (67.4% vs. 42.7%) showed a significantly higher prevalence of QQ (Table 3).

A logistic regression analysis (Table 5) was conducted to identify independent associations of QQ among nurses. The overall model demonstrated a good fit (Hosmer‐Lemeshow *χ* ^2^ = 6.242, *df* = 8, *p* = 0.62) and correctly classified 67.8% of cases, explaining 19.8% of the variance in QQ (Nagelkerke *R*‐squared = 0.198). The logistic regression analysis revealed that hospital type, job satisfaction, and burnout were significant predictors of QQ. Specifically, working in a specialized hospital was associated with a significantly higher odds of QQ (OR = 2.249, 95% CI [1.581, 3.199], *p* < 0.001). Conversely, higher job satisfaction was associated with a significantly lower odds of QQ (OR = 0.747, 95% CI [0.586, 0.954], *p* = 0.019). Higher burnout levels were associated with a significantly higher odds of QQ (OR = 1.49, 95% CI [1.3, 1.709], *p* < 0.001). Additionally, higher monthly working hours were marginally associated with increased odds of QQ (OR = 1.003; 95% CI: 1.000–1.007; *p* = 0.05). Age, sex, education class, total clinical experience, family or friend engagement, job tenure in current department, experiencing violence, having thought of emigration, and job turnover intention were not significantly associated with QQ in this model.

**TABLE 5:** Logistic regression analysis predicting quiet quitting.

Independent variables	Adjusted beta coefficient	B 95% CI for beta (lower‐upper)	*p*‐value
Age (years)	−0.053	0.95 (0.89–1.01)	0.087
Sex (male vs. female)	0.352	1.42 (0.96–2.11)	0.080
Education class (bachelor and higher degree vs. master or lower educations)	0.352	1.42 (0.80–2.50)	0.226
Total clinical experience (years)	0.038	1.04 (0.97–1.11)	0.243
Hospital type (specialized vs. general hospital)	0.810	2.25 (1.58–3.20)	**< 0.001**
Monthly work hours	0.003	1.00 (1.00–1.01)	0.050
Family or friend engagement (no vs. yes)	0.335	1.40 (0.82–2.38)	0.218
Job tenure in current department (years)	0.143	1.15 (0.80–1.66)	0.439
Experiencing violence (yes vs. no)	0.121	1.13 (0.81–1.58)	0.480
Having thought of emigration (yes vs. no)	0.161	1.18 (0.84–1.66)	0.336
Job turnover intention (yes vs. no)	0.029	1.03 (0.70–1.51)	0.884
Job satisfaction	−0.291	0.75 (0.59–0.95)	**0.019**
Burnout	0.399	1.49 (1.30–1.71)	**< 0.001**

p‐value is less than 0.05 (p < 0.05).

## 4 Discussion

In this cross‑sectional study, we found a high prevalence of QQ among Iranian nurses, with burnout, hospital type, and job satisfaction emerging as the main associated factors.

We found that 59.5% of nurses met the cut‑off for QQ (2.38), with a mean total QQ score of 2.50 ± 0.55, indicating that more than half of the sample reported moderate levels of disengagement.

In a study of Greek nurses, the prevalence of QQ was 67% using a lower cut‑off of 2.06 and a lower mean total score of 2.27 ± 0.66 (Galanis et al. 2024). These differences in prevalence and mean scores may relate to methodological differences different cut‑off points, as well as broader economic, cultural, and organizational contexts between Iran and Greece.

Examining the QQ subscales revealed that a lack of motivation had the highest average QQ score, while a lack of initiation was in second place, and mean score of detachment subscale was lower than the others. This finding with the exact order resonates with prior research (Galanis et al. 2024; Toska et al. 2025); for example, in Greek nurses, figures were as follows: 2.96 for lack of motivation, 2.35 for lack, and 2.07 for detachment (Galanis et al. 2024). As previous studies have found in order to improve lack of motivation and initiation, customized reward systems (Toska et al. 2025), professional development opportunities, or even strategies to enhance nurses' sense of self‐efficacy and control over their work, could prove effective in combating QQ (Moisoglou et al. 2025).

In this study, detachment subscale yielded the lowest scores, which may be hypothesized to reflect nurses’ awareness of the high‐stakes nature of their profession (Alami et al. 2024). The potential for severe repercussions—including legal or professional sanctions—in cases of negligence or inadequate patient care could act as a deterrent and may help explain why nurses do not actively disengage to the point of jeopardizing patient safety (Jackson et al. 2010; Ghahramani et al. 2021). Overall, this interpretation should be viewed as a hypothesis rather than a conclusion, and the low detachment scores do not necessarily imply high engagement but may reflect pragmatic professional boundaries and perceived consequences.

In the logistic regression model, nurses working in specialized hospitals had more than twice the odds of QQ compared with those in general hospitals. This contrasts with an Ethiopian study on burnout, where nurses in primary hospitals were more susceptible to burnout than those in tertiary hospitals (Yisa et al. 2024). One plausible hypothesis is that the intense, high‐pressure environments of specialized hospitals, coupled with limited resources, restricted autonomy, or a lack of variety in tasks, are associated with greater burnout and disengagement, which in turn could contribute to higher levels of QQ. But current study is the first study that has evaluated association of QQ among nurses and specialty of hospital, and further research is needed.

In the current study, longer monthly working hours were associated with higher QQ; however, this relationship was only borderline (*p* = 0.05) and should be interpreted cautiously. Prior research among healthcare staff in Nigeria suggests that excessive workload is a major contributor to QQ and job dissatisfaction (Yisa et al. 2024). Importantly, monthly working hours may be an imperfect proxy for workload because it does not capture workload intensity or staffing conditions. In our study, the measure assessing “adequate time to spend with friend/family” is more indicative of work–life balance than workload per se; notably, 89% of nurses reported insufficient time for social and family life. Future studies should therefore assess workload using more direct indicators (e.g., patient‐to‐nurse ratio, overtime frequency, shift intensity, staffing adequacy, and task burden) in addition to working hours.

In our study, burnout was a strong predictor of QQ, whereas higher job satisfaction showed a protective effect. These associations are consistent with prior research showing that burnout is directly and indirectly linked to QQ through mental health and turnover intentions and that job satisfaction and organizational support can buffer this effect (Galanis et al. 2024; Payscale 2023; Gün et al. 2025; Ghahramani et al. 2023). For example, a study of 383 nurses reported that job satisfaction and organizational support mediated the relationship between burnout and QQ (Gün et al. 2025). Together, these findings highlight the importance of creating supportive work environments that reduce burnout and enhance satisfaction through recognition, opportunities for professional growth, teamwork, and involvement in decision‑making (Geng et al. 2025; Karadas and Çevik 2025).

In the current study, nurses with fewer than 10 years of experience reported a higher prevalence of QQ compared with those with ≥ 10 years of experience (65% vs. 49.8%). Consistent with this pattern, Alai et al., in a machine‐learning study of QQ among healthcare staff, also identified years of experience as an important factor associated with QQ (Alami et al. 2024). This finding has practical significance because it identifies early‐career nurses as a higher risk group for QQ, supporting the need for targeted onboarding, mentorship, and early supportive interventions.

Experience of workplace violence was a strong predictor of QQ. One plausible pathway is that exposure to violence contributes to burnout and job dissatisfaction, which may in turn increase the likelihood of QQ. Supporting this, another study highlighted workplace bullying as a specific form of violence associated with QQ among nurses and suggested it may be mitigated through effective coping strategies (Galanis et al. 2024). In other words, when workplace violence is high, QQ can be a hidden signal that the system is “paying its costs” through silence and withdrawal.

Notably, while there was a high prevalence of QQ among nurses with job turnover intention among participants (63.7% compared to 49.3%), this did not emerge as a significant independent predictor of QQ in logistic regression analysis. This finding contrasts with earlier studies (Galanis et al. 2023; Gün et al. 2025), indicating that although many nurses consider leaving their jobs, their actual engagement levels are more significantly affected by immediate job satisfaction and burnout rather than their intention to depart. Another explanation might be higher levels of resilience among these nurses; however, this remains a hypothesis, as we did not assess resilience in the present study (Ghahramani et al. 2023).

Thoughts of emigration may stem from burnout, dissatisfaction, and other significant predictors, as well as societal and political factors that were not assessed in this study; however, these mechanisms remain speculative and should be explored in future research.

### 4.1 Strengths and Limitations of the Study

In contrast to many prior QQ studies that used online self‑administered questionnaires, we collected data through face‑to‑face interviews at the workplace.

We chose this approach because we hypothesized that nurses experiencing QQ might be less willing to engage with non‑essential online tasks, potentially reducing response rates in web‑based surveys (Galanis et al. 2024). While some nurses might have viewed participation as an unwelcome job‐related activity, the interview format also offered an opportunity for engagement and a temporary respite from routine. As a result, this approach is controversial, but we assume it can enhance participation and ensure a more accurate assessment of QQ. Previous studies have typically relied on online questionnaires and smaller samples, for example, surveys of 186 healthcare professionals in hospital settings (Ghahramani et al. 2025; Lalkhen and McCluskey 2008), whereas our study included 754 nurses across 13 hospitals in two cities. (Toska et al. 2025; Geng et al. 2025).

Selecting the Galanis QQ Scale (Galanis et al. 2023), despite the existence of alternatives like the 14‐item Turkish version QQ and QF (Karadas and Çevik 2025), was a conscious decision rooted in its design specifically as a QQ scale. QQ questionnaire, which was developed and validated by Galanis et al., had three subscales—lack of motivation, lack of initiative, and detachment—offered a richer, more nuanced lens through which to understand the multifaceted nature of QQ. The Turkish version of QQ and QF scale (Karadas and Çevik 2025) was adopted from another questionnaire that was initially designed for a broader employee population (Anand et al. 2024). Also this scale evaluates QF (quiet firing) which was not goal of our study.

Several limitations should be considered when interpreting the findings. This study was conducted on self‐reported data. While this method is often used in social science research, it introduces the potential for social desirability bias. We did not investigate all possible contributing factors, such as specific aspects of organizational culture or individual personality traits that may also play a role in influencing QQ behaviors among nurses. Notably, low salary has been identified as a significant motivational factor contributing to QQ behaviors, like a study in Nigeria (Yisa et al. 2024); however, our study was limited to examining this single factor and did not encompass the broader context of financial compensation. Additionally, our sample was restricted to nurses operating within a specific region of Iran, which may limit the generalizability of our findings to the entire Iranian nursing population. While we collected data on several potential correlates of QQ, we acknowledge that our exploration was not exhaustive. Also, the QQ among nurses working in private hospitals, primary healthcare centers, and outpatient clinics was not assessed.

## 5 Conclusion

The most important finding of this study is that more than half of studied nurses reported to having QQ. The findings underscore the urgent need for healthcare organizations to address the fundamental issues contributing to this phenomenon, including burnout, job dissatisfaction, and high work hours, particularly in specialized hospitals. Moreover, it is essential for healthcare leaders to prioritize initiatives that recognize and address the unique challenges faced by nurses, thereby creating an atmosphere conducive to professional fulfillment and job satisfaction. Future research should focus on evaluating the effectiveness of various interventions designed to combat QQ and improve overall patient outcomes.

## Author Contributions

**Hassan Gholami**: formal analysis, software. **Sulmaz Ghahramani**: conceptualization, methodology, writing – review and editing, software, supervision, visualization. **Ahmad Nemati**: conceptualization, methodology, data curation, project administration, writing – original draft, writing – review and editing, visualization. **Mahdi Hazrati**: data curation, validation, investigation, writing – original draft. **Aida Eskandari**: data curation, investigation. **Kamran Bagheri Lankarani**: supervision, conceptualization, resources. **Mobin Gholami**: writing – original draft, validation, formal analysis, software.

## Ethics Statement

Ethical approval for this study was conducted by the Ethics Committee of the Research Department at Shiraz University of Medical Sciences (IR.SUMS.REC.1402.583) and in accordance with relevant guidelines and regulations. Also, it was conducted in accordance with the Declaration of Helsinki. All nurses provided informed consent and were assured that their personal information would be kept confidential. They were also informed of their right to withdraw from the study at any time.

## Funding

The authors have nothing to report.

## Conflicts of Interest

The authors declare no conflicts of interest.

## Supporting information




**Supplementary Materials**: brb371439‐sup‐0001‐SuppMat.docx

## Data Availability

The data supporting the findings of this study can be obtained from the corresponding author upon reasonable request.
