# Supplementary material for: Quiet Quitting of Job in Nurses Working in Hospitals, a Multicenter Study in Iran
Source: Brain Behav. 2026 Apr 29;16(5):e71439. doi: 10.1002/brb3.71439 (PMC13128962; doi:10.1002/brb3.71439)
Supplement: Supplementary file 1 — Supplementary Materials: brb371439‐sup‐0001‐SuppMat.docx [file BRB3-16-e71439-s001.docx]

**Quiet Quitting of job in nurses working in hospitals, A multi-center study in Iran**

**Methods:**

**Validity and Reliability Assessment**

In this study, we used the 9-item Quiet Quitting Scale (QQS) developed by Galanis and colleagues, which has demonstrated acceptable validity and reliability in previous studies conducted outside Iran. (1) Psychometric properties, including face validity, content validity, concurrent validity, and reliability, were evaluated for the QQS.

A panel of eight experts assessed the face validity of the QQS by evaluating the relevance, clarity, and difficulty of each item.

Content validity was assessed by a group of ten experts with backgrounds in epidemiology, biostatistics, nursing practice, hospital management, head nursing, community medicine, and public health. The Content Validity Index (CVI) was used to evaluate the relevance of the questions based on a four-point scale (1 = not relevant, 2 = needs essential revision, 3 = relevant but needs revision, and 4 = very relevant). The CVI was calculated by dividing the number of respondents who scored three or four by the total number of respondents. A score below 0.70 indicates that the item is unacceptable and should be removed.

We assessed concurrent validity using the Job Satisfaction Survey (JSS), the single item burnout measure, and a single-item measure for job turnover intention. We calculated 1-specificity, sensitivity, and Youden's J index ([sensitivity+specificity]−1), which indicates the overall effectiveness of both sensitivity and specificity. Values closer to 1 indicate greater effectiveness. (2)

Receiver operating characteristic (ROC) curves, illustrating sensitivity versus (1-specificity), were used to determine the optimal trade-off between sensitivity and specificity compared with a reference standard. (3) We calculated the area under the ROC curve (AUROC) with a 95% confidence interval (CI) to compare the overall performance of the QQS. Statistical tests within the ROC Curve framework evaluated whether the model possesses statistically significant discriminatory power and whether the observed differences between models are non-random. In the context of burnout, job satisfaction, and intention to leave, these tests examined the extent to which the QQS score effectively differentiates nurses experiencing these conditions from those who do not, thereby assessing its validity as a predictive measure in occupational settings.

Internal consistency reliability was measured using Cronbach's alpha, with an alpha value of 0.6 or higher considered acceptable.

**Results**

The QQS demonstrated acceptable psychometric properties. In the face validity assessment, expert opinions were collected, and none of the items were removed. The CVI score in the content validity assessment was 0.89, indicating acceptable validity.

The 9-item Quiet Quitting Scale showed an AUROC of 0.61 (0.56-0.65) for job turnover intention, 0.69 (0.64-0.74) for burnout, and 0.67 (0.64-0.71) for job satisfaction, all with P < 0.001, indicating a significantly high performance. Cronbach’s alpha for the QQS was 0.66.

Sensitivities, specificities, and Youden's J indices for different tools are displayed in Supplementary Table 1. According to Youden's J index, 2.38 was the optimal cutoff for detecting Quiet Quitting (QQ) in the QQS. However, Youden’s J index should be interpreted with caution, as sensitivity and specificity are equally weighted in its calculation. When using QQS, sensitivity and specificity are not always regarded as equally important. It appears that sensitivity is generally prioritized over specificity (4, 5). Therefore, we considered 2.38 as the cutoff point for identifying nurses who are quiet quitters.

Table S1. Performance of 9 items QQS for detecting burn out, job satisfaction and job turn over intention (N= 754)

| Criterion | Cut- off | Sensitivity | 1-Specificity | Youden's J index |
| --- | --- | --- | --- | --- |
| Job turnover intention | 2.38 | 0.637 | 0.493 | 0.507 |
| Burn out | 2.38 | 0.654 | 0.323 | 0.677 |
| Job satisfaction | 2.38 | 0.674 | 0.427 | 0.573 |

**References**

1. Galanis P, Katsiroumpa A, Vraka I, Siskou O, Konstantakopoulou O, Moisoglou I, et al. The quiet quitting scale: Development and initial validation. AIMS public health. 2023;10(4):828-48.

2. Youden WJ. Index for rating diagnostic tests. Cancer. 1950;3(1):32-5.

3. Wallace LS, Rogers ES, Roskos SE, Holiday DB, Weiss BD. Brief report: screening items to identify patients with limited health literacy skills. Journal of general internal medicine. 2006;21:874-7.

4. Lalkhen AG, McCluskey A. Clinical tests: sensitivity and specificity. Continuing education in anaesthesia, critical care & pain. 2008;8(6):221-3.

5. Barnard‐Brak L, Brewer A, Chesnut S, Richman D, Schaeffer AM. The sensitivity and specificity of the social communication questionnaire for autism spectrum with respect to age. Autism Research. 2016;9(8):838-45.

Table 1. Characteristics of study participants (N=547)

| Variable | Subgroups | Frequency | Percent |
| --- | --- | --- | --- |
| Age group | 18-45 | 415 | 75.9 |
|  | 46-65 | 132 | 24.1 |
| Gender | Male | 208 | 38.0 |
|  | Female | 339 | 62.0 |
| City | Shiraz | 302 | 55.2 |
|  | Hamadan | 245 | 44.8 |
| Job-status | Employed | 253 | 46.3 |
|  | Unemployed (or housekeeper) | 294 | 53.7 |
| Education | Under the diploma | 123 | 22.5 |
|  | Diploma or higher | 424 | 77.5 |
| Participants status | Outpatient | 179 | 32.7 |
|  | Inpatient | 178 | 32.5 |
|  | Healthy | 190 | 34.7 |
| Health Literacy Level based on the HELIA questionnaire | Inadequate | 56 | 10.2 |
|  | Marginal | 136 | 24.9 |
|  | Adequate | 234 | 42.8 |
|  | Excellent | 121 | 22.1 |

Table 2. Areas under the receiver operating characteristic (AUROC) and 95% confidence interval (CI) for each of the screening questions (N=547)

| Screening Questions | Inadequate Health Literacy | Limited Health Literacy |
| --- | --- | --- |
| Help Read | 0.588 (0.511-0.666) | 0.589 (0.54-0.639) |
| Problems Reading | 0.629 (0.549-0.709) | 0.635 (0.587-0.683) |
| Confident with Forms | 0.635 (0.559-0.712) | 0.627 (0.578-0.676) |
| Comprehension of Prescriptions | 0.690 (0.620-0.759) | 0.666 (0.619-0.713) |

Table 3. Areas under the receiver operating characteristic (AUROC) and 95% confidence interval (CI) for different combinations of screening questions (N=547)

| Screening Questions | Inadequate Health Literacy | Limited Health Literacy |
| --- | --- | --- |
| All four questions | 0.7 (0.629-0.771) | 0.683 (0.636-0.729) |
| Chew screening questions | 0.66 (0.584-0.737) | 0.657 (0.61-0.704) |
| Help Read & Problems Reading | 0.634 (0.555-0.713) | 0.629 (0.581-0.677) |
| Help Read & Confident with Forms | 0.634 (0.559-0.71) | 0.629 (0.581-0.678) |
| Help Read & Comprehension of Prescriptions | 0.67 (0.6-0.74) | 0.651 (0.603-0.698) |
| Problems Reading & Confident with Forms | 0.668 (0.594-0.742) | 0.667 (0.621-0.714) |
| Confident with Forms & Comprehension of Prescriptions | 0.705 (0.638-0.773) | 0.674 (0.627-0.72) |
| Problems Reading & Comprehension of Prescriptions | 0.695 (0.624-0.766) | 0.682 (0.636-0.727) |

Table 4. Performance of screening questions for detecting inadequate health literacy (N=547)

| Question | Criterion | Sensitivity | Specificity | Youden's J index | +LR (95% CI) | -LR (95% CI) |
| --- | --- | --- | --- | --- | --- | --- |
| Help Read | ≥ Never | 100 | 0 | 0 | 1 |  |
|  | ≥ Occasionally | 83.93 | 31.16 | 0.15 | 1.22 (1.07 - 1.39) | 0.52 (0.28 - 0.95) |
|  | ≥ Sometimes | 53.57 | 54.58 | 0.08 | 1.18 (0.91 - 1.53) | 0.85 (0.63 - 1.14) |
|  | ≥ Often | 28.57 | 78.41 | 0.06 | 1.32 (0.85 - 2.07) | 0.91 (0.77 - 1.08) |
|  | ≥ Always | 21.43 | 92.06 | 0.13 | 2.7 (1.50 - 4.84) | 0.85 (0.74 - 0.98) |
| Problems Reading | ≥ Never | 100 | 0 | 0 | 1 |  |
|  | ≥ Occasionally | 89.29 | 16.29 | 0.05 | 1.07 (0.97 - 1.18) | 0.66 (0.30 - 1.44) |
|  | ≥ Sometimes | 73.21 | 46.64 | 0.19 | 1.37 (1.15 - 1.64) | 0.57 (0.37 - 0.89) |
|  | ≥ Often | 42.86 | 75.36 | 018 | 1.74 (1.24 - 2.44) | 0.76 (0.60 - 0.96) |
|  | ≥ Always | 19.64 | 92.87 | 0.12 | 2.76 (1.48 - 5.11) | 0.87 (0.76 - 0.99) |
| Confident with Forms | ≤ Extremely | 100 | 0 | 0 | 1 |  |
|  | ≤ Quite a bit | 82.14 | 37.68 | 0.19 | 1.32 (1.15 - 1.52) | 0.47 (0.27 - 0.84) |
|  | ≤ Somewhat | 48.21 | 68.43 | 0.16 | 1.53 (1.13 - 2.06) | 0.76 (0.58 - 0.98) |
|  | ≤ A little bit | 23.21 | 91.04 | 0.14 | 2.59 (1.49 - 4.51) | 0.84 (0.73 - 0.98) |
|  | ≤ Not at all | 8.93 | 97.15 | 0.06 | 3.13 (1.17 - 8.37) | 0.94 (0.86 - 1.02) |
| Comprehension of Prescriptions | ≤ Extremely | 100 | 0 | 0 | 1 |  |
|  | ≤ Quite a bit | 85.71 | 40.94 | 0.26 | 1.45 (1.27 - 1.65) | 0.35 (0.18 - 0.67) |
|  | ≤ Somewhat | 58.93 | 73.73 | 0.32 | 2.24 (1.72 - 2.92) | 0.56 (0.41 - 0.77) |
|  | ≤ A little bit | 17.86 | 90.63 | 0.08 | 1.91 (1.02 - 3.56) | 0.91 (0.80 - 1.03) |
|  | ≤ Not at all | 3.57 | 97.15 | 0 | 1.25 (0.29 - 5.37) | 0.99 (0.94 - 1.05) |

Table 5. Performance of screening questions for detecting limited health literacy (N=547)

| Question | Criterion | Sensitivity | Specificity | Youden's J index | +LR (95% CI) | -LR (95% CI) |
| --- | --- | --- | --- | --- | --- | --- |
| Help Read | ≥ Never | 100 | 0 | 0 | 1 |  |
|  | ≥ Occasionally | 80.21 | 34.93 | 0.15 | 1.23 (1.11 - 1.37) | 0.57 (0.41 - 0.78) |
|  | ≥ Sometimes | 53.65 | 57.75 | 0.11 | 1.27 (1.06 - 1.52) | 0.8 (0.67 - 0.96) |
|  | ≥ Often | 26.56 | 80 | 0.06 | 1.33 (0.97 - 1.82) | 0.92 (0.83 - 1.01) |
|  | ≥ Always | 14.58 | 93.52 | 0.08 | 2.25 (1.33 - 3.80) | 0.91 (0.86 - 0.97) |
| Problems Reading | ≥ Never | 100 | 0 | 0 | 1 |  |
|  | ≥ Occasionally | 92.19 | 20 | 0.12 | 1.15 (1.08 - 1.23) | 0.39 (0.23 - 0.66) |
|  | ≥ Sometimes | 69.27 | 52.11 | 0.21 | 1.45 (1.25 - 1.67) | 0.59 (0.47 - 0.75) |
|  | ≥ Often | 36.46 | 78.87 | 0.15 | 1.73 (1.31 - 2.27) | 0.81 (0.71 - 0.91) |
|  | ≥ Always | 13.54 | 94.37 | 0.07 | 2.4 (1.38 - 4.19) | 0.92 (0.86 - 0.97) |
| Confident with Forms | ≤ Extremely | 100 | 0 | 0 | 1 |  |
|  | ≤ Quite a bit | 76.56 | 42.25 | 0.18 | 1.33 (1.18 - 1.49) | 0.55 (0.42 - 0.74) |
|  | ≤ Somewhat | 45.31 | 73.24 | 0.18 | 1.69 (1.34 - 2.14) | 0.75 (0.65 - 0.86) |
|  | ≤ A little bit | 16.15 | 92.68 | 0.08 | 2.2 (1.35 - 3.60) | 0.9 (0.84 - 0.97) |
|  | ≤ Not at all | 5.21 | 97.46 | 0.02 | 2.05 (0.85 - 4.97) | 0.97 (0.94 - 1.01) |
| Comprehension of Prescriptions | ≤ Extremely | 100 | 0 | 0 | 1 |  |
|  | ≤ Quite a bit | 79.69 | 47.89 | 0.27 | 1.53 (1.35 - 1.73) | 0.42 (0.31 - 0.57) |
|  | ≤ Somewhat | 45.31 | 78.87 | 0.24 | 2.14 (1.66 - 2.77) | 0.69 (0.60 - 0.80) |
|  | ≤ A little bit | 14.06 | 91.83 | 0.05 | 1.72 (1.05 - 2.82) | 0.94 (0.88 - 1.00) |
|  | ≤ Not at all | 1.56 | 96.34 | -2.1 | 0.43 (0.12 - 1.48) | 1.02 (0.99 - 1.05) |

Figure 1. Receiver operating characteristic (ROC) curves of four screening questions to identify inadequate (A) and limited (B) health literacy


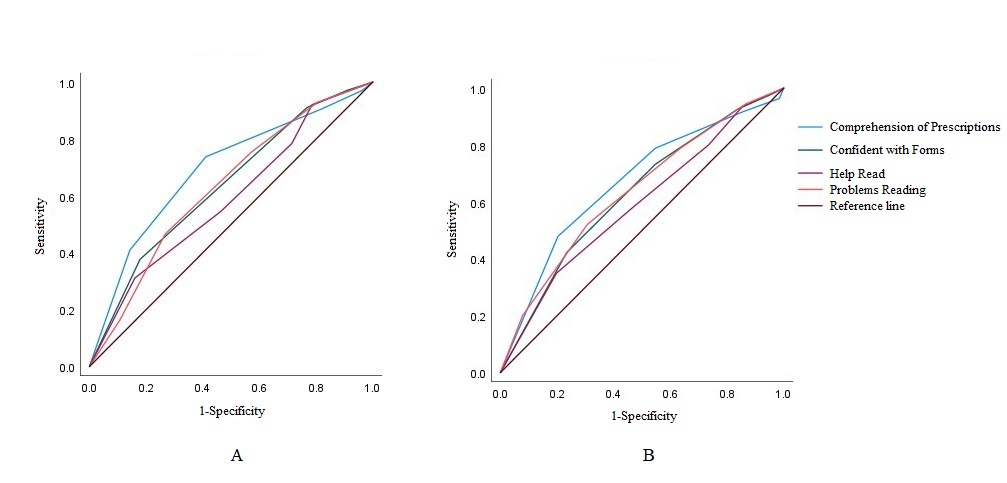


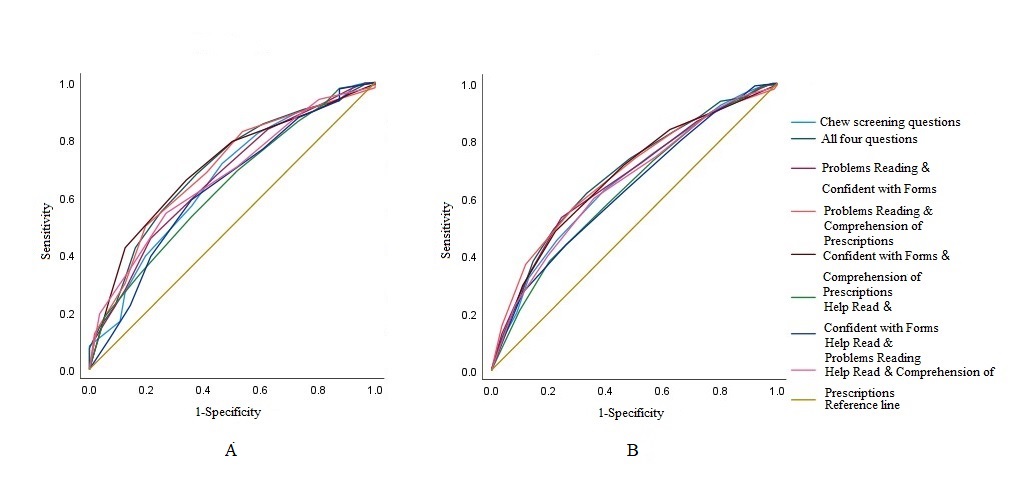


Figure 2. Receiver operating characteristic (ROC) curves of different combinations of screening questions to identify inadequate (A) and limited (B) health literacy
